# Body mass index, waist circumference, and waist-to-height ratio for prediction of multiple metabolic risk factors in Chinese elderly population

**DOI:** 10.1038/s41598-017-18854-1

**Published:** 2018-01-10

**Authors:** Zhan Gu, Dong Li, Huayu He, Jianying Wang, Xiaojuan Hu, Peihua Zhang, Yanlong Hong, Baocheng Liu, Lei Zhang, Guang Ji

**Affiliations:** 1 Shanghai Innovation Center of TCM Health Service, Shanghai University of Traditional Chinese Medicine, No. 1200 Cailun Road, Shanghai, 201203 China; 2 Zhangjiang Community Health Service Center of Pudong New District, No. 458 Yijiang Road, Shanghai, 201210 China; 3 Institute of Digestive Diseases, China-Canada Center of Research for Digestive Diseases (ccCRDD), Longhua Hospital, Shanghai University of Traditional Chinese Medicine, No. 725 South Wanping Road, Shanghai, 200032 China

## Abstract

The purpose of this study was to compare the predictive ability of five obesity indices, including body mass index (BMI), waist circumference (WC), waist-to-height ratio (WHtR), waist-to-hip ratio (WHpR) and body adiposity index (BAI), to predict multiple non-adipose metabolic risk factors, including elevated blood pressure (BP), elevated fasting plasma glucose (FPG), elevated triglyceride (TG), reduced high-density lipoprotein cholesterol (HDL-C), elevated serum uric acid (SUA) and non-alcoholic fatty liver disease (NAFLD), in an elderly Chinese population. A total of 5685 elderly Chinese subjects (≥60 years) were recruited into our community-based cross-sectional study. Receiver operating characteristic curve (ROC) analyses were used to compare the predictive ability as well as determine the optimal cut-off values of the obesity indices for multiple metabolic risk factors. According to the areas under the receiver operating characteristic curve (AUC), BMI, WC and WHtR were able to similarly predict high metabolic risk in males (0.698 vs. 0.691 vs. 0.688), while in females, BMI and WC were able to similarly predict high metabolic risk (0.676 vs. 0.669). The optimal cut-off values of BMI, WC and WHtR in males were, respectively, 24.12 kg/m^2^, 83.5 cm and 0.51, while in females, the values were 23.53 kg/m^2^ and 77.5 cm.

## Introduction

Metabolic syndrome (MetS) is a complex disease spectrum with a clustering of metabolic and cardiovascular risk factors^1^. MetS has a high general prevalence of 33.9% among Chinese adults^2^. According to the diagnosis criteria proposed by the International Diabetes Federation (IDF) and the American Heart Association (AHA)/National Heart, Lung and Blood Institute (NHLBI) in 2009^3^, obesity, elevated blood pressure (BP), elevated fasting plasma glucose (FPG), elevated triglyceride (TG) and reduced high-density lipoprotein cholesterol (HDL-C) are the main components of MetS. In addition to these factors, numerous studies support the hypothesis that elevated serum uric acid (SUA)^4–7^ and non-alcoholic fatty liver disease (NAFLD)^8–10^ are also risk factors for MetS. Uric acid is the end-product of purine metabolism in humans, and an altered SUA level is associated with glucose and lipid metabolism^11,12^. In numerous studies, hyperuricemia is considered to be a risk factor for MetS or metabolic disorders^4–7^. NAFLD is a clinicopathological condition characterized by abnormal lipid deposition in hepatocytes^13^ and has been suggested to be a hepatic manifestation of MetS^14,15^. Therefore, obesity, elevated BP, elevated FPG, elevated TG, reduced HDL-C, elevated SUA and NAFLD are considered to be the common and important metabolic risk factors for MetS in our study.

Obesity is already a serious public health burden worldwide^16^. Up to 50% of elderly Chinese have an abnormally high body mass index, causing a large disease-related healthcare burden^17^. Various obesity-related anthropometric indices, such as body mass index (BMI), waist circumference (WC), waist-to-height ratio (WHtR), waist-to-hip ratio (WHpR) and body adiposity index (BAI), have been used to predict metabolic risk factors in numerous studies^18–22^. BMI is a measurement of body fat based on height and weight^23^, while WC reflects abdominal or central obesity. WHtR and WHpR further reflect the fat distribution based on WC, and all three are considered to be specific alternatives to assess abdominal fat^24^. In contrast to all of the previous indicators, BAI is a recent indicator and is used to estimate the amount of body fat^25,26^.

The purpose of this study is to compare the predictive ability of these obesity indices for predicting multiple non-adipose metabolic risk factors in elderly Chinese subjects at or above 60 years of age. To further evaluate the importance of obesity factor in Chinese elderly people, we adopted the five commonly used obesity indices mentioned above as well as more comprehensive metabolic risk factors, including elevated BP, elevated FPG, elevated TG, reduced HDL-C, elevated SUA and NAFLD, in the present study.

## Results

### Baseline characteristics of study subjects

As shown in Table 1, there were no significant statistical differences by gender for age and BMI. In contrast, WC and WHpR were greater in males compared to females, while female subjects had greater WHtR and BAI compared to males (*P* value < 0.001). Among the multiple metabolic risk factors, the prevalence of elevated BP, elevated FPG and NAFLD did not differ by gender. The prevalence of elevated TG and reduced HDL-C was greater in females compared to males, while male subjects had an increased prevalence of elevated SUA compared to females (*P* value < 0.001).

**Table 1:** Baseline characteristics of study subjects. Data are expressed as mean± standard deviation, median (interquartile range 25–75%), or counts (percentages). WC: waist circumference; BMI: body mass index; WHtR: waist-to-height ratio; WHpR: waist-to-hip ratio; BAI: body adiposity index; SBP: systolic blood pressure; DBP: diastolic blood pressure; FPG: fasting plasma glucose; TG: triglyceride; HDL-C: high-density lipoprotein cholesterol; SUA: serum uric acid; BP: blood pressure; NAFLD: non-alcoholic fatty liver disease.

Variable	Total (n = 5685)	Male (n = 2543)	Female (n = 3142)	*P* value
Age (years)	70.16 ± 7.50	70.05 ± 7.27	70.24 ± 7.67	0.333
Height (cm)	161.23 ± 7.62	167.36 ± 5.55	156.26 ± 5.02	<0.001
Weight (kg)	63.14 ± 10.96	68.14 ± 10.33	59.10 ± 9.73	<0.001
WC (cm)	81.70 ± 9.19	83.41 ± 8.99	80.32 ± 9.12	<0.001
BMI (kg/m^2^)	24.22 ± 3.43	24.29 ± 3.23	24.16 ± 3.59	0.175
WHtR	0.51 ± 0.06	0.50 ± 0.05	0.51 ± 0.06	<0.001
WHpR	0.88 ± 0.06	0.89 ± 0.06	0.86 ± 0.06	<0.001
BAI	27.63 ± 4.07	25.26 ± 3.06	29.54 ± 3.77	<0.001
SBP (mmHg)	138.45 ± 22.40	138.62 ± 22.06	138.32 ± 22.67	0.621
DBP (mmHg)	82.19 ± 12.39	81.66 ± 12.66	82.63 ± 12.16	0.003
FPG (mmol/L)	5.6(5.2–6.3)	5.6(5.2–6.4)	5.6(5.2–6.2)	0.011
TG (mmol/L)	1.28(0.94–1.83)	1.21(0.86–1.72)	1.36(1.01–1.91)	<0.001
HDL–C (mmol/L)	1.25(1.07–1.44)	1.16(1.02–1.35)	1.31(1.13–1.50)	<0.001
SUA (μmol/L)	320.2(271.1–378.3)	356.0(304.5–410.3)	294.7(252.4–343.7)	<0.001
Elevated BP	3902 (68.64%)	1728 (67.95%)	2174 (69.19%)	0.316
Elevated FPG	2997 (52.72%)	1376 (54.11%)	1621 (51.59%)	0.059
Elevated TG	1688 (29.69%)	660 (25.95%)	1028 (32.72%)	<0.001
Reduced HDL-C	2218 (39.01%)	711 (27.96%)	1507 (47.96%)	<0.001
Elevated SUA	764 (13.44%)	559 (21.98%)	205 (6.52%)	<0.001
NAFLD	2409 (42.37%)	1036 (40.74%)	1373 (43.70%)	0.025

### Number of metabolic risk factors and obesity indices

Table 2 shows the association of metabolic risk factors and obesity indices. BMI, WC, WHtR, WHpR and BAI showed an increase in the number of metabolic risk factors in both genders. As shown in Table 2, 2653 elderly subjects (1115 men and 1538 women) had ≥3 metabolic risk factors. We estimated that approximately half of the elderly subjects (46.67% in total, 43.85% in males and 48.95% in females) in our study had high metabolic risk.

**Table 2:** Number of metabolic risk factors and obesity indices.

N (Risk factors) N (Male/Female)	0 (199/199)	1 (515/573)	2 (714/832)	3 (558/779)	4 (363/482)	5 (163/256)	6 (31/21)	≥3 (1115/1538)
**BMI**								
Male	21.90 ± 2.78	22.88 ± 2.94	24.16 ± 3.20	25.00 ± 2.99	25.85 ± 2.88	25.83 ± 2.62	26.86 ± 2.81	25.45 ± 2.93
Female	21.74 ± 3.04	22.68 ± 3.38	23.79 ± 3.49	24.68 ± 3.35	25.44 ± 3.21	26.45 ± 3.53	26.11 ± 3.07	25.23 ± 3.39
**WC**								
Male	76.90 ± 7.47	79.46 ± 8.08	82.93 ± 8.39	85.46 ± 8.82	87.88 ± 8.36	87.38 ± 7.45	91.35 ± 8.11	86.70 ± 8.57
Female	73.85 ± 7.71	76.58 ± 8.81	79.58 ± 8.68	81.80 ± 8.76	82.97 ± 7.92	86.14 ± 9.01	86.24 ± 5.95	82.95 ± 8.65
**WHtR**								
Male	0.46 ± 0.05	0.48 ± 0.05	0.50 ± 0.05	0.51 ± 0.05	0.52 ± 0.05	0.52 ± 0.05	0.55 ± 0.05	0.52 ± 0.05
Female	0.47 ± 0.05	0.49 ± 0.06	0.51 ± 0.06	0.52 ± 0.06	0.53 ± 0.05	0.55 ± 0.06	0.55 ± 0.04	0.53 ± 0.06
**WHpR**								
Male	0.86 ± 0.06	0.87 ± 0.05	0.89 ± 0.06	0.90 ± 0.06	0.92 ± 0.05	0.91 ± 0.05	0.94 ± 0.06	0.91 ± 0.06
Female	0.83 ± 0.06	0.84 ± 0.06	0.86 ± 0.06	0.87 ± 0.06	0.88 ± 0.05	0.90 ± 0.06	0.90 ± 0.04	0.88 ± 0.06
**BAI**								
Male	23.54 ± 2.97	24.48 ± 2.90	25.14 ± 2.85	25.69 ± 3.01	26.30 ± 3.04	26.24 ± 3.10	26.96 ± 3.41	26.00 ± 3.06
Female	27.65 ± 3.42	28.65 ± 3.63	29.24 ± 3.82	30.00 ± 3.70	30.39 ± 3.37	30.89 ± 3.97	30.72 ± 3.44	30.28 ± 3.66

### AUC of obesity indices for multiple metabolic risk factors

Table 3 shows the areas under the receiver operating characteristic curve (AUC) for BMI, WC, WHtR, WHpR and BAI for each multiple metabolic risk factors in both genders using receiver operating characteristic curve (ROC) analyses. BMI had the strongest predictive ability for elevated BP, elevated FPG and NAFLD in both genders; for the elevated SUA of males; and for the elevated TG of females. WC had the strongest predictive ability for reduced HDL-C of both genders as well as for the elevated TG of males. WHtR had the strongest predictive ability for the elevated SUA of females.

**Table 3:** Area under curves (95% CI) of obesity indices for metabolic risk factors. *P value < 0.05.

	BMI	WC	WHtR	WHpR	BAI
**Male**					
Elevated BP	0.626 (0.603–0.649)*	0.613 (0.590–0.636)*	0.623 (0.600–0.646)*	0.593 (0.569–0.616)*	0.606 (0.583–0.629)*
Elevated FPG	0.612 (0.590–0.634)*	0.600 (0.578–0.622)*	0.598 (0.576–0.620)*	0.597 (0.575–0.619)*	0.562 (0.540–0.585)*
Elevated TG	0.647 (0.624–0.670)*	0.648 (0.625–0.671)*	0.642 (0.619–0.666)*	0.630 (0.606–0.654)*	0.592 (0.567–0.616)*
Reduced HDL–C	0.637 (0.614–0.660)*	0.649 (0.627–0.672)*	0.645 (0.622–0.668)*	0.626 (0.603–0.650)*	0.593 (0.569–0.617)*
Elevated SUA	0.581 (0.555–0.608)*	0.578 (0.553–0.604)*	0.577 (0.551–0.603)*	0.572 (0.546–0.597)*	0.553 (0.526–0.579)*
NAFLD	0.638 (0.616–0.659)*	0.631 (0.610–0.653)*	0.624 (0.603-0.646)*	0.605 (0.583–0.628)*	0.586 (0.563–0.608)*
**Female**					
Elevated BP	0.578 (0.557–0.600)*	0.569 (0.547–0.590)*	0.570 (0.548––0.592)*	0.557 (0.535-0.578)*	0.554 (0.532–0.576)*
Elevated FPG	0.598 (0.578–0.618)*	0.591 (0.572–0.611)*	0.584 (0.564–0.603)*	0.566 (0.546–0.586)*	0.561 (0.541–0.581)*
Elevated TG	0.615 (0.595–0.635)*	0.601 (0.581–0.621)*	0.590 (0.570–0.611)*	0.576 (0.555–0.597)*	0.563 (0.542–0.584)*
Reduced HDL–C	0.621 (0.601–0.640)*	0.632 (0.612–0.651)*	0.622 (0.603–0.642)*	0.615 (0.595–0.634)*	0.576 (0.556–0.596)*
Elevated SUA	0.615 (0.575–0.654)*	0.641 (0.602–0.679)*	0.651 (0.614–0.688)*	0.630 (0.590–0.669)*	0.628 (0.590–0.666)*
NAFLD	0.656 (0.637–0.675)*	0.653 (0.634–0.672)*	0.641 (0.621–0.660)*	0.618 (0.598–0.638)*	0.596 (0.576–0.616)*

.

**Figure 1:**
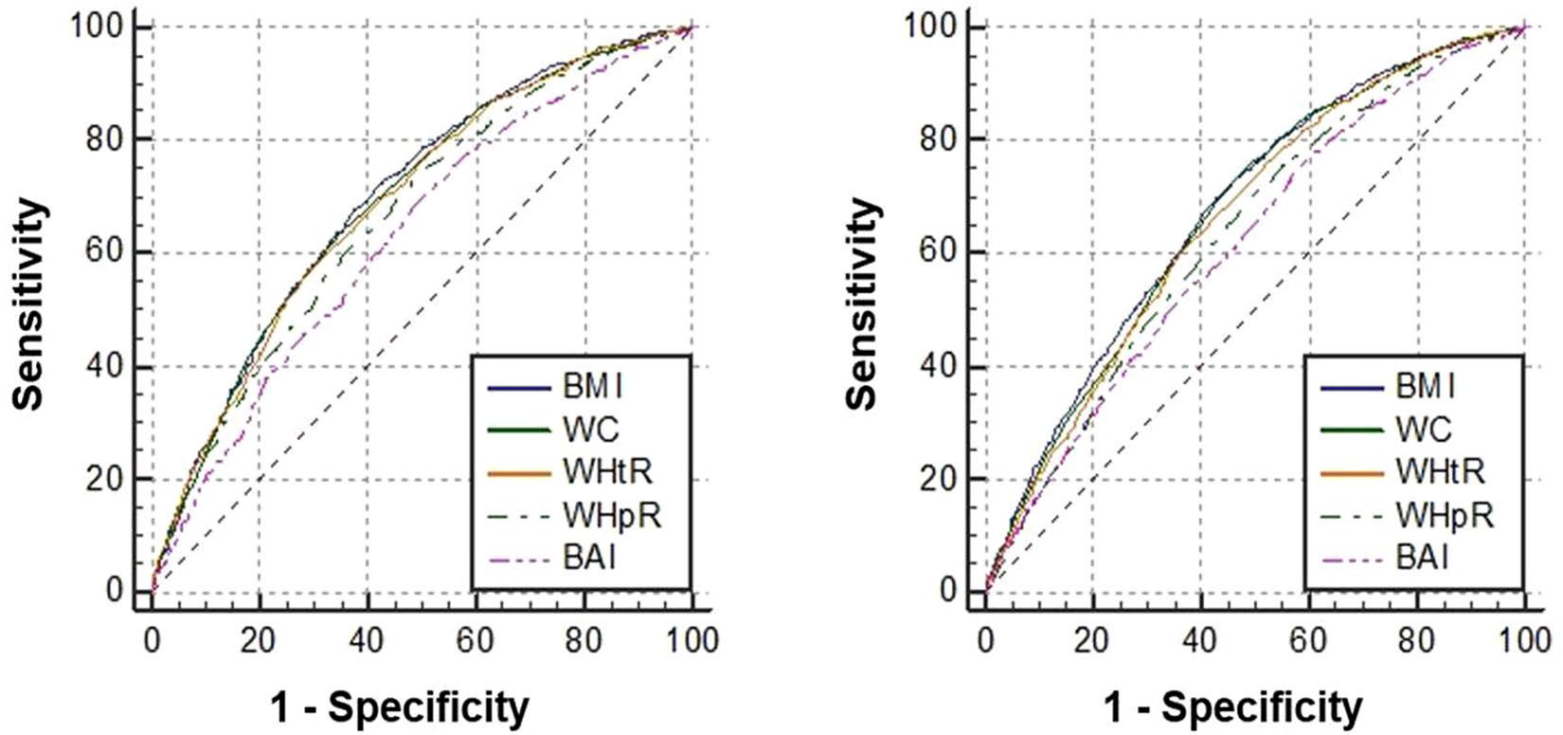
ROC curves of obesity indices to predict high metabolic risk population. Male (**left**) and female (**right**).

ROC analyses were applied to compare the predictive ability of obesity indices to predict the presence of a high metabolic risk population that had ≥3 non-adipose metabolic risk factors. Figure 1 shows the ROC curves for the five obesity indices to predict high metabolic risk in males and females. The AUC, optimal cut-off values, sensitivity, specificity and Youden index of the five obesity indices for predicting high metabolic risk are shown in Table 4. BMI had the strongest predictive ability for both genders. The statistical significance of the AUC differences of the five obesity indices suggested that the AUC of BMI, WC and WHtR did not differ in males and were all greater than WHpR and BAI; however, in females, the AUC of BMI and WC did not differ and were greater than for WHtR, WHpR and BAI. WHpR and BAI had a low predictive ability in both genders. The optimal cut-off values of BMI, WC and WHtR in males were 24.12 kg/m^2^, 83.5 cm and 0.51, respectively, while in females, the values were 23.53 kg/m^2^ and 77.5 cm.

**Table 4:** AUC, optimal cut-off values, sensitivity, specificity and Youden index of obesity indices to predict the high metabolic risk population. *P value < 0.05. **The obesity indices were able to similarly predict ≥3 metabolic risk factors using MedCalc statistical software with the algorithm developed by DeLong’s research team.

	AUC (95%CI)	Cut-off value	Sensitivity (%)	Specificity (%)	Youden index (%)
**Male**					
BMI	0.698 (0.677–0.718)**	24.12	67.9	62.5	30.4
WC	0.691 (0.671–0.712)**	83.5	63.9	64.6	28.5
WHtR	0.688 (0.668–0.709)**	0.51	56.2	71.6	27.9
WHpR	0.666 (0.646–0.687)*	0.88	72.8	51.7	24.5
BAI	0.629 (0.608–0.651)*	24.47	69.4	50.6	20.0
**Female**					
BMI	0.676 (0.657–0.694)**	23.53	70.0	57.1	27.1
WC	0.669 (0.650–0.688)**	77.5	76.3	50.2	26.5
WHtR	0.659 (0.640–0.678)*	0.49	76.4	47.4	23.8
WHpR	0.632 (0.613–0.652)*	0.85	71.0	49.6	20.6
BAI	0.614 (0.595–0.634)*	27.82	74.9	42.3	17.2

## Discussion

In this study, we found BMI, WC and WHtR to be more accurate than WHpR and BAI for predicting multiple metabolic risk factors; BMI, WC and WHtR were able to similarly predict the high metabolic risk population in males, while in females, BMI and WC were able to similarly predict the high metabolic risk population.

Obesity plays a central role in MetS and is closely associated with various metabolic risk factors. In addition to the five main components of MetS, we used elevated SUA and NAFLD as two additional metabolic risk factors in this study as there are numerous studies indicating that elevated SUA and NAFLD are risk factors for MetS and metabolic disorders^4–10^. To the best of our knowledge, this is one of the first comprehensive studies to evaluate the predictive ability of obesity indices with multiple metabolic risk factors and to focus on a Chinese elderly population. The strengths of our study include the sample size, the specificity of our population, and the additional use of elevated SUA and NAFLD risk factors.

The results showed that BMI and WC were more useful for predicting multiple metabolic risk factors, in line with Wang *et al*.’s research^19^. We also found that WHtR had the same predictive ability as BMI and WC for predicting the high metabolic risk population in males. A European study suggested that BMI, WC and WHtR were associated with clustered cardiometabolic risk factors and that the magnitudes of the associations were similar for BMI, WC and WHtR using mixed-effect regression and ROC analyses^27^. Liu *et al*. have found that the BMI, WC and WHtR values were able to equivalently predict the presence of multiple metabolic risk factors using ROC analyses in a general Chinese adult population^21^. Our results validated these findings in elderly males, while we found that BMI and WC had more significant predictive value than WHtR in elderly females. Moreover, we found that both WHpR and BAI in either gender was a much weaker predictor, supporting Yu *et al*.’s research^28^.

In the present study, the optimal cut-off values of the appropriate obesity indices were determined to predict the high metabolic risk population according to gender among the Chinese elderly. The BMI values for both genders were approximately 24 kg/m^2^, in line with Wang *et al*.’s research in north China^19^, and were higher than Liu *et al*.’s research, which was conducted in a general adult population in northeast China^21^ (22.85 and 23.30 kg/m^2^ in males and females). The WC values in both genders were lower than in Wang *et al*.’s results^19^ (88.05 and 87.10 cm in males and females aged 65–85 years), Liu *et al*.’s results^21^ (91.3 and 87.1 cm in males and females) and Guan *et al*.’s results conducted in Chinese rural adults^18^ (85 and 80 cm in males and females). The WHtR value in males was in line with Liu *et al*.’s results^21^ and was higher than that of Guan *et al*.’s results^18^ (0.50 in males). Wang *et al*.’s research suggested that the obesity indices for the prediction of MetS changed with age distribution^19^. Our study focuses on elderly Chinese people aged 60 years and above in Shanghai, and the differences in the results may be due to the age and regional distributional differences of the samples. As these obesity indices change with age and gender^29^, “one for all” cut-off values of obesity indices for all age groups are not appropriate to assess multiple metabolic risk factors.

There are, however, several limitations of this study. The subjects in this study were all from Shanghai, and the sample may not fully reflect all Chinese elderly. This study included quite a large number of Chinese elderly, but we only enrolled subjects who had completed the comprehensive health check study, which may have biased our primary findings. Further studies with multi centers and a larger sample size are needed to identify the association of obesity indices with more comprehensive metabolic risk factors.

## Methods

### Study subjects

This study is a community-based cross-sectional investigation for the elderly population in Shanghai, China. A total of 5736 residents from Zhangjiang community of Shanghai aged  ≥60 years were recruited into our study between April and July 2015. The study was performed according to the guidelines of the Helsinki Declaration. A standard protocol was designed by Shanghai innovation center of TCM health service and was approved by the Ethics Committee of Shanghai University of Traditional Chinese Medicine. Written informed consents were obtained from all subjects.

The inclusion criteria included age ≥ 60 years, local residents in Shanghai, complete data measurements and informed consents. Subjects with mental disorders, malignant tumors or incomplete recorded information were excluded from this project based on their medical records. After investigation, 51 subjects were excluded from the study. A total of 5685 Chinese elderly subjects (2543 males and 3142 females) with complete data were finally included in this study.

### Data measurements

The anthropometric indices included WC, hip circumference (HC), height and weight. WC and HC were measured to the nearest 0.1 cm using a flexible metric measuring tape (Pudong CDC; Shanghai, China). Height and weight were measured to the nearest 0.1 cm and 0.1 kg using electronic measurement instrument (Shengyuan; Zhengzhou, China). All subjects were measured wearing light clothing without hats and shoes. BMI was calculated as bodyweight (kg)/height^2^ (m^2^). WHtR was calculated as WC (cm)/height (cm). WHpR was calculated as WC (cm)/HC (cm). BAI was calculated as HC (cm)/height^1.5^ (m) minus 18^26^.

Blood pressure was measured with electronic sphygmomanometers (Biospace; Cheonan, South Korea) using the standard recommended procedures. Blood samples were obtained from the antecubital vein in the morning after an overnight fasting period. FPG, TG, HDL-C and SUA were measured using an automatic biochemistry analyzer (Hitachi; Tokyo, Japan). The color ultrasound system (TOSHIBA; Tokyo, Japan) was used by two experienced radiologists to screen for fatty liver.

### Criteria for multiple metabolic risk factors and the “high metabolic risk population”

According to the diagnosis criteria of MetS in 2009^3^, the multiple non-adipose metabolic risk factors included the following items: (a) elevated BP: systolic blood pressure (SBP) ≥ 130 mmHg or diastolic blood pressure (DBP) ≥ 85 mmHg, or ongoing antihypertensive medications; (b) elevated FPG: FPG ≥ 5.6 mmol/L, or ongoing anti-diabetic treatment; (c) elevated TG: TG ≥ 1.7 mmol/L; and (d) reduced HDL-C: HDL-C < 1.04 mmol/L in males and HDL-C < 1.30 mmol/L in females. In addition to the regular MetS components, we also enrolled: (e) elevated SUA: SUA > 420 μmol/L according to the diagnosis criteria of hyperuricemia in 2000^30^; (f) NAFLD: no history of heavy drinking, liver ultrasound imaging for diffuse fatty liver, and no drug-induced liver disease or viral hepatitis according to the diagnosis criteria of NAFLD in 2012^31^. Subjects with three or more of the six non-adipose metabolic risk factors were defined as the “high metabolic risk population” in present study.

### Statistical analyses

All of the descriptive statistics for all of the variables were calculated. Continuous variables were expressed as mean ± standard deviation and compared using two-sided *t* tests. Categorical variables were expressed as counts or percentages and compared using Pearson’s *χ* ^2^ tests. To compare the predictive ability and determine the optimal cut-off values of the obesity indices for predicting multiple metabolic risk factors, ROC analyses were used. AUCs were calculated, and the optimal cut-off values were identified from the maximum Youden index (sensitivity plus specificity-1) to determine the appropriate obesity indices.

*P* values < 0.05 were set as significant for all of the statistical tests for bilateral contrasts. All statistical analyses were conducted using SPSS version 17.0 (SPSS; Chicago, USA). The statistical significance of the differences in the AUCs was analyzed using MedCalc version 17.1.0 (MedCalc; Ostend, Belgium) with the algorithm developed by DeLong’s research team^32^.
